# Combination of Molecular Networking and LC-MS/MS Profiling in Investigating the Interrelationships between the Antioxidant and Antimicrobial Properties of *Curculigo latifolia*

**DOI:** 10.3390/plants10081488

**Published:** 2021-07-21

**Authors:** Nadiah Mad Nasir, Nur Syafiqah Ezam Shah, Nurul Zulaikha Zainal, Nur Kartinee Kassim, Siti Munirah Mohd Faudzi, Hanan Hasan

**Affiliations:** 1 Department of Chemistry, Faculty of Science, Universiti Putra Malaysia (UPM), Serdang 43400, Selangor, Malaysia; syafiqahezam1995@gmail.com (N.S.E.S.); zulaikhazainal5@gmail.com (N.Z.Z.); kartinee@upm.edu.my (N.K.K.); sitimunirah@upm.edu.my (S.M.M.F.); 2 Institute Bioscience, Universiti Putra Malaysia (UPM), Serdang 43400, Selangor, Malaysia; 3 Department of Food Science, Faculty of Food Science and Technology, Universiti Putra Malaysia (UPM), Serdang 43400, Selangor, Malaysia; mhanan@upm.edu.my

**Keywords:** molecular networking, *Curculigo latifolia*, antioxidant activity, antibacterial activity, free radical scavenging, total phenolic content, β-carotene bleaching assay

## Abstract

*Curculigo* is a potent plant with a variety of traditional uses, such as anti-oxidant, anti-diabetic, anti-tumor, anti-bacterial, anti-cancer, anti-osteoporosis, and wound-healing. The comprehensive profiling of the *Curculigo* *latifolia* metabolome was carried out by generating a molecular network (MN) from Liquid Chromatography–Tandem Mass Spectrometry (LC-MS/MS) data to profile the methanol extract and correlating them with their antioxidant (2,2-diphenyl-1-picryl-hydrazyl-hydrate (DPPH), total phenolic contents (TPC), and β-carotene) and antimicrobial (disk-diffusion agar method, minimum inhibitory concentration (MIC), and minimum bactericidal concentration (MBC)) properties. The antioxidant capacity was observed to be significantly higher in the rhizome crude extract, with 18.10 ± 0.91 µg/mL DPPH activity, and a β-carotene bleaching result of 35.20%. For the antimicrobial activity, the leaf crude extract exhibited a strong *Staphylococcus aureus* and *Salmonella choleraesuis* (8–15 ± 3.0 mm) inhibition in the disk-diffusion agar. The leaf extract also exhibited maximum antibacterial activity against *S. aureus* (MIC = ±0.25 mg/mL, MBC = ±0.25 mg/mL) and *S. choleraesuis* (MIC = ±0.25 mg/mL, MBC = ±0.25 mg/mL). LC-MS/MS analysis and MN revealed norlignans and phenolic glycosides as major metabolites in the rhizome and leaf extracts of the negative mode (M − H)^−^. Fourteen known compounds were identified, and three unknown compounds were putatively identified in the rhizome extract, while ten known compounds and six unknown compounds were putatively identified in the leaf extract.

## 1. Introduction

*Curculigo latifolia,* commonly known as ‘lemba’, is a perennial plant belonging to the Hypoxidaceae family that originates from Malaysia. This plant is well known for its sweet proteins, namely curculin and neoculin, which have been proven to be 500 to 9000 times sweeter than sucrose by weight [1]. This species is propagated via rhizomes [2] and are abundantly found in highland areas (1500–2000 m altitude), mostly on slopes and in forests. The leaf fibers can be used to make fishing nets, ropes, and twine, and are predominantly utilized in Borneo and Malaysia [3]. The leaves, stem-tips, and rhizomes of *C. latifolia* have been used domestically as traditional medicine to combat fever [4]. Decoctions of the flowers and roots are used as stomachic and diuretic [5], whereas the rhizome is used to treat menorrhagia and is applied in traditional medicine as a lotion against ophthalmia [4]. In Borneo, the leaves of *C. latifolia* are used in traditional healing ceremonies [4]. The rhizomes of the plant have also been used as a traditional cure for jaundice, while the rhizome extract of *C. latifolia* has been shown to inhibit the hepatitis B virus in vitro, which further reinforces the plant’s usefulness as a traditional medicine [6].

The biological effects exhibited by the *Curculigo* species with *Curculigo orchioides* originating from China and India being the most famous—ensures that it is frequently used to treat piles, asthma, jaundice, colic, diarrhea, and gonorrhea [7]. Moreover, according to Murali and Kuttan [8], the rhizomes of *C. orchioides* have strong antioxidant properties that are attributed to the presence of a phenolic derivative (Curculigoside). Furthermore, *C. orchioides* leaf extracts have also exhibit antimicrobial activity, whereby the maximum zone of growth inhibition (agar well diffusion) was observed against *Pseudomonas aeruginosa* and *Staphylococcus aureus* [9]. *P. aeruginosa* is capable of causing acute and chronic infections at different sites within the body such as the urinary tract, the skin (via burns or surgical wounds), and the respiratory tract [10], while *S. aureus* is a major human pathogen that causes a wide range of clinical infections [11].

*Curculigo pilosa* (Africa) is another famous *Curculigo* species that is traditionally used as anti-epileptic medication, to increase fertility, for meteorism relief, as a styptic, and for drepanocytosis management. It is also an antibacterial, antioxidant, and antidiabetic agent [12,13,14,15]. *C. pilosa* has been shown to exhibit antioxidant activity, with the crude extract having the highest total phenolic content (TPC), total flavonoid content (TFC), and total antioxidant capacity (TAC), indicating a greater DPPH scavenging effect [12]. 

Many *Curculigo* plant products contain norlignans, triterpenoids, and phenol glycosides as major metabolites, of which are essential for plant development and play an important role in their defense mechanisms. The norlignans constitute a large class of natural phenolic compounds that exhibit powerful biological activity which may serve as lead compounds, for example, for the development of anticancer agents [16]. 

Phenolic glycosides play important biological roles, including being responsible for stimulating the immune response by acting on macrophages and lymphocytes [17]. Previous studies on the rhizomes of *C. orchioides* (Hypoxidaceae) have highlighted the potent antioxidative and antiosteoporotic activities that are present in phenolic glycosides [18,19,20]. Furthermore, Durazzo [21] reported that the determination and measurement of antioxidant content can bring about health benefits. The understanding and assessment of the interactions between these biologically active compounds and other food matrix components could be viewed as the main step in the investigation of total antioxidant properties. However, because of their biological role and various modes of action, it is difficult to assess a single and efficient procedure for evaluating antioxidant activity. Hence, in the evaluation of antioxidant properties, three essential elements should be identified and developed: the extraction procedure, antioxidant capacity measurements, and the expression of results [22]. 

The literature studies reported here show that the *Curculigo* plant has many benefits; hence, the objective of this study was to comprehensively profile selected parts of *C. latifolia* via molecular networking (MN) from the Liquid Chromatography–Tandem Mass Spectrometry (LC-MS/MS) data obtained from the methanol extract. The dereplication information was then collected by the MN, which then compares the MS/MS spectra of the investigated compounds and groups them into clusters based on their fragmentation route similarities. MN is used to visualize the structural link between molecules belonging to the same molecular family, making it easier to identify unknown metabolites [23]. The resulting data was then correlated with the plant’s antioxidant and antimicrobial activities, which could potentially help the pharmaceutical industry to further develop the plant for the treatment of various diseases.

## 2. Results and Discussion

### 2.1. Metabolite Profiling Determined via LC-MS/MS

**Figure 1:**
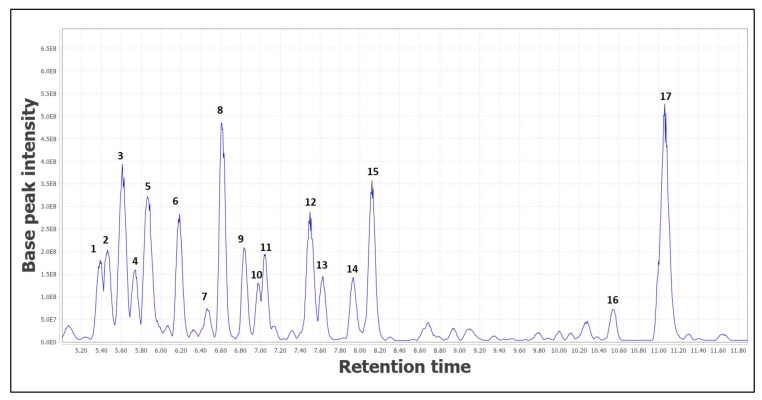
Total ion chromatogram (TIC) in negative ion mode of the rhizome extract. The number above each peak represents the peak numbers, corresponding to the peak numbers in Table 1.

**Figure 2:**
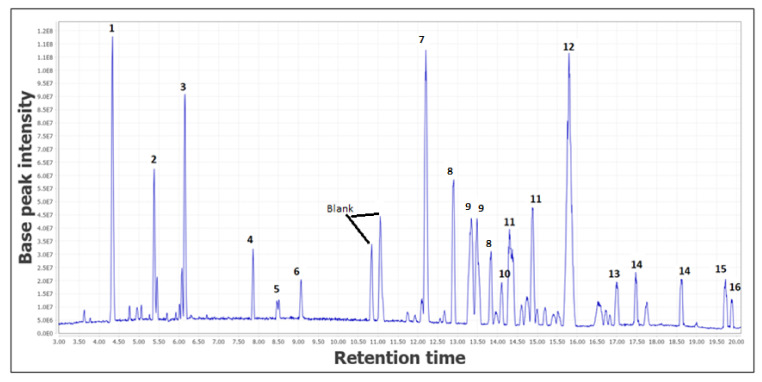
Total ion chromatogram (TIC) in negative ion mode of the leaf extract. The number above each peak represents the peak numbers, corresponding to the peak numbers in Table 2.

The metabolite profiling of the *C. latifolia* rhizome and leaf extract was carried out via LC-MS/MS. The total scan Photodiode-Array Detection (PDA) chromatogram and the total ion chromatogram (TIC) of the extracts are shown in Figure 1 and Figure 2, respectively. A total of 17 chromatographic peaks were annotated in the rhizome extract and 16 chromatographic peaks were annotated in the leaf extract. Generally, the compound classes contained in the extract were firstly obtained from the ultraviolet (UV) absorptions of the compound peaks observed in the total scan PDA chromatograms. Most of the UV-absorbing compounds detected by the PDA detector showed maximum absorptions in the 200–300 nm range. Supplementary Figures S1–S6 highlight the identities of the metabolites that were elucidated based on their molecular masses and mass fragmentation patterns. The LC-MS analysis was run in positive and negative modes, but most of the compounds were better ionized in the negative mode. Therefore, the compounds were identified based on their full MS and MS/MS spectra obtained in the negative ion mode. The LC-MS/MS revealed the diverse norlignans and phenolic glycosides compounds found in the rhizome and leaf extracts. The rhizome and leaf extracts were chosen based on their highest antioxidant and antimicrobial activity, correspondingly.

### 2.2. Molecular Network

**Figure 3:**
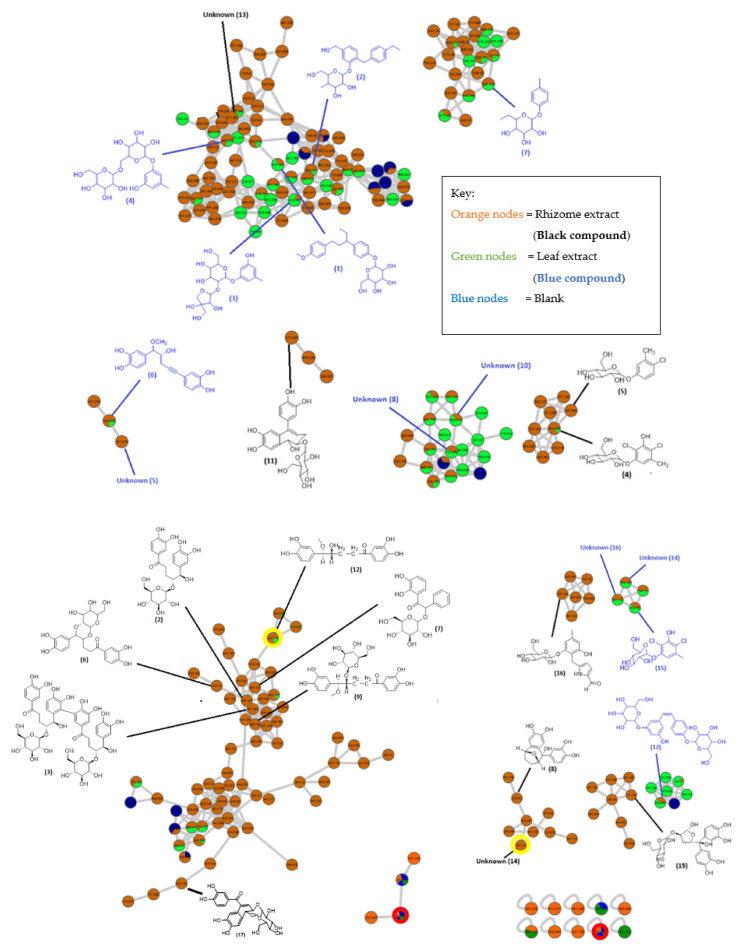

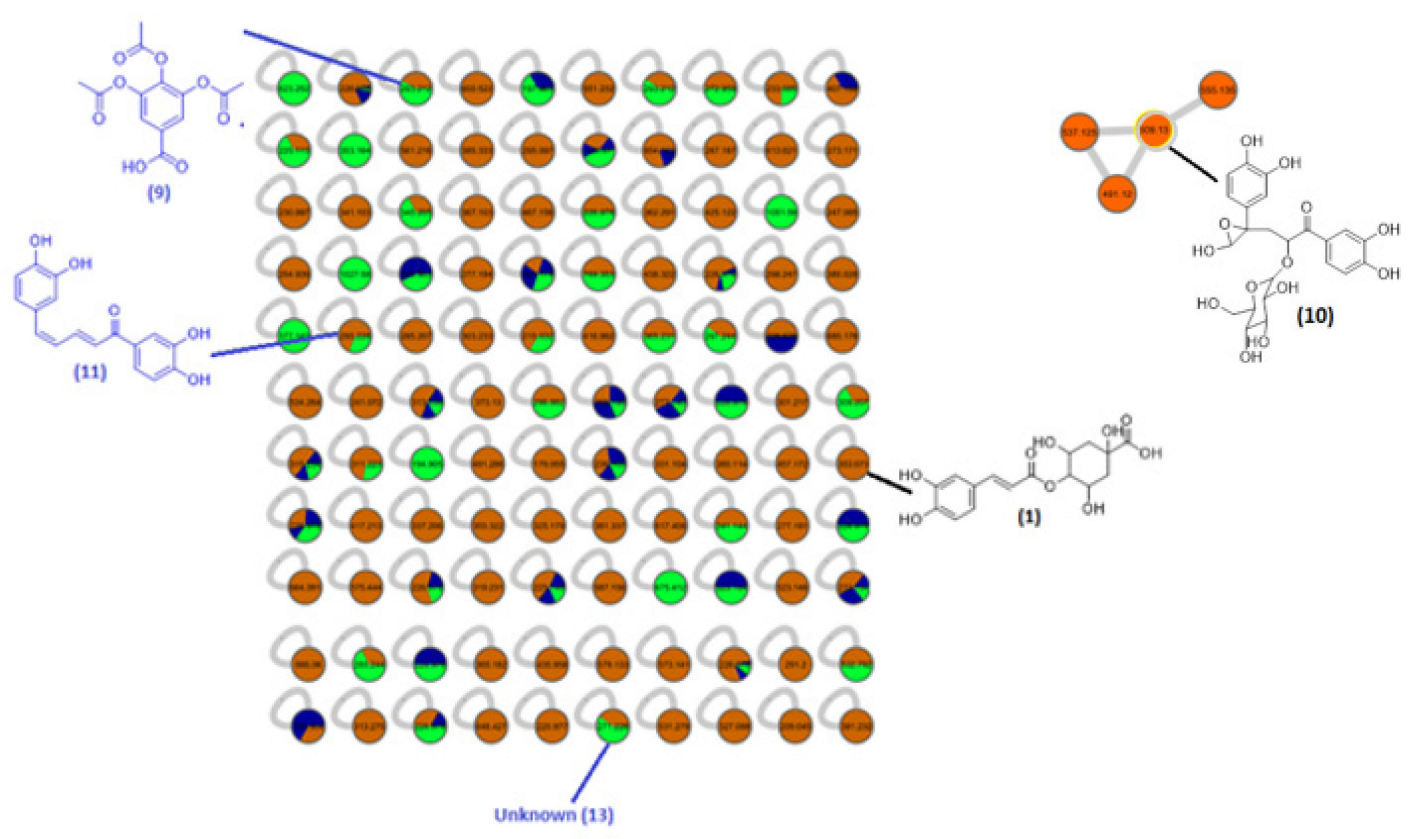
Annotation of the molecular networking of the norlignans and phenolic glycoside derived from *Curculigo* *latifolia* rhizome and leaf extracts, obtained from the GNPS library and PubChem platforms. The black colored structures (**---**) are from the rhizome extract and blue color structures (**---**) are from the leaf extract.

MN facilitates data mining via the clustering of the MS/MS spectra based on fragmentation cosine similarities [24]. The MN of the rhizome and leaf extract were generated from the LC-MS/MS analysis data to analyze the metabolite content of *C. latifolia* more comprehensively and accurately. Figure 3 shows the generated MN from the rhizome and leaf extracts with different clusters, whereby each cluster shares some distinct fragments and fragmentation patterns. The putative annotation was conducted with reference to the different mass spectroscopic databases that are available.

Two major and distinct clusters were putatively identified in the network comprising of clusters of norlignans and phenolic glycosides. The metabolites were identified based on a systematic study of the fragmentation pathways and patterns observed from the resulting network. The mass spectrometry-based MN allows for the identification and putative annotation of 17 metabolites in the rhizome crude and 16 metabolites in the leaf crude, as shown in Table 1 and Table 2. The MN was capable of not only dereplicating known metabolites, but also help point out related derivatives, of which are described for the first time in *C. latifolia*. 

#### 2.2.1. Metabolite Profiling of the Rhizome Extract Based on LC-MS/MS and MN

**Figure 4:**
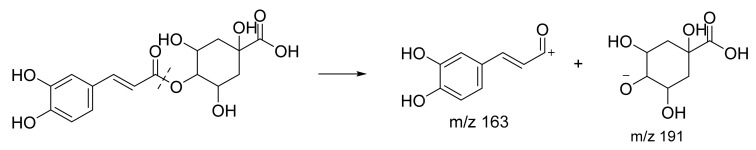
Proposed fragmentation pathway for 4-[[3-(3,4- dihydroxyphenyl)-1-oxo-2-propenyl]oxy]-1,3,5-trihydroxycyclohexanecarboxylic acid.

In the rhizome extract, 14 known compounds and three unknown compounds were identified (black colored structures). The norlignans and intermediate lignin were recognizable based on their mass fragmentation pathway, as shown in Table 1 (Figure 1) and all the detected compounds were compared with published data [25,26,27,28,29,30,31,32,33,34,35,36,37,38]. Among all the peaks in the LC-MS/MS spectra, only peak 1 represented an intermediate lignin, consistent with 4-[[3-(3,4-ihydroxyphenyl)-1-oxo-2-propenyl]oxy]-1,3,5 trihydroxycyclohexanecarboxylic acid. The parent ion at 353 [M − H]^−^ and the peak occurred at RT 5.39, resulting in MS/MS fragment ions at *m*/*z* 191.0553 (the loss of *m*/*z* 163 was due to the elimination of 1,3,4,5-tetrahydroxycyclohexane-1-carboxylic acid from the parent ion). A possible fragmentation pathway for 4-[[3-(3,4-dihydroxyphenyl)-1-oxo-2-propenyl]oxy]-1,3,5-trihydroxycyclohexanecarboxylic acid is shown in Figure 4, and is in line with published work [27] (Supplementary Figure S1).

**Figure 5:**
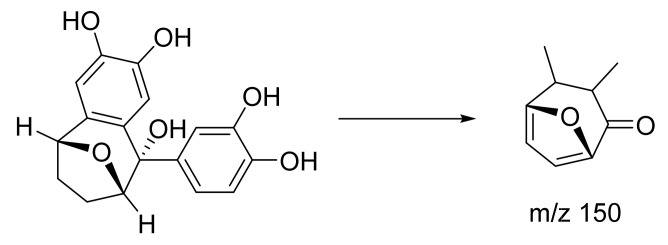
Proposed fragmentation pathway for sinensigenin A.

Peak 8 with RT 6.63 was identified as sinensigenin A (Table 1) and was the most abundant among the norlignans in the rhizome extract with a parent ion at *m*/*z* 315 [M − H]^−^ (Figure 5). The parent ion further dissociated to give fragment ions at *m*/*z* 150, showing a loss of two aromatic rings from sinensigenin A. While this compound was previously identified in the rhizomes of *Curculigo* *sinensis* [30], it is the first time that the compound is identified in *C. latifolia* (Supplementary Figure S2).

**Figure 6:**
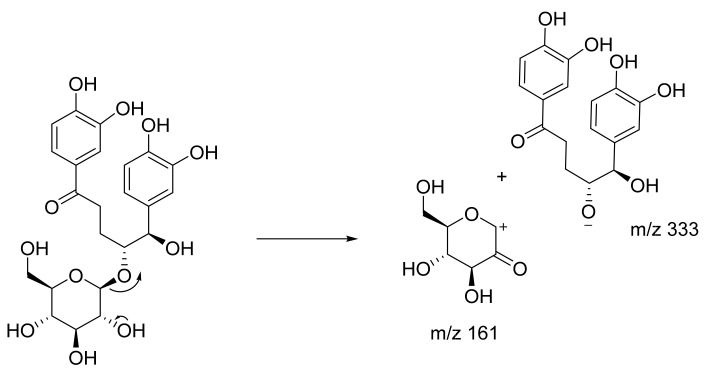
Proposed fragmentation pathway for curculigine.

**Figure 7:**
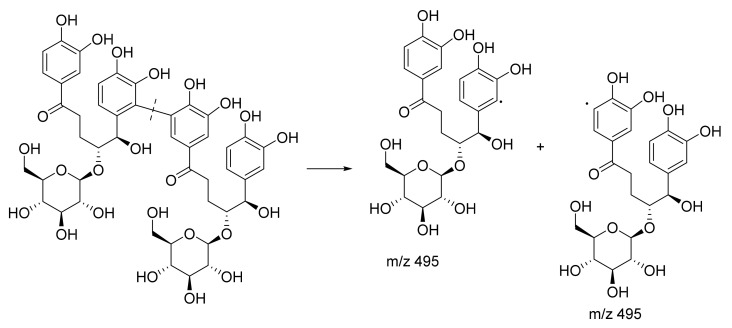
Proposed fragmentation pathway for the dimer of curculigine.

Among the three unknown compounds, one unknown compound was putatively identified as a dimer of curculigine, as the mass value was double that of the known curculigine (Figure 6, Supplementary Figure S3). The dimer of curculigine has a parent ion at 991 [M − H]^−^ and occurred at RT 5.61, resulting in MS/MS fragment ions at *m*/*z* 495.1506 (the loss of *m*/*z* 495 was due to the elimination of curculigine from the parent ion). A possible fragmentation pathway for the dimer of curculigine is shown in Figure 7. Furthermore, this result is supported by the MN (Figure 3), whereby the mass of the curculigine dimer (peak 3) is located in the same cluster as the mass of curculigine (peak 2). This suggests that both the compounds have the same skeleton structure.

Interestingly, from the LC-MS/MS data, two peaks showed the same mass value of *m*/*z* 347 (peaks 12 and 14 in Table 1). Initially it was thought that this could be attributed to two compounds with diastereomer structures. However, when viewed in the MN, the *m*/*z* 347 mass appeared in two different clusters. From the MN, it can be concluded that peak 12 and peak 14 are two different compounds with different skeletons, although they both share the same mass value (as circled in yellow color in Figure 3). Peak 12 was identified as curculigenin with an ion parent at 347 [M − H]^−^, seeing that it was located in the same cluster as curculigine (peak 2), curcapicycloside (peak 6), and 1-*O*-methylisocurculigine (peak 9). All these compounds showed that curculigenins clustered per their substituent’s similarities. Based on the MN, another unknown compound (peak 14) with a mass value of *m*/*z* 347 [M − H]^−^ was deduced to be a derivative of the sinensigenin A (peak 8) compound because this unknown compound is located in the same cluster as sinensigenin A (*m*/*z* 315 [M − H]^−^) as shown in Figure 3. In the MN of the rhizome extract, one major cluster which consists of six known norlignans was annotated. Curculigine (peak 2), curcapicycloside (peak 6), 1-(2,3-Dihydroxyphenyl)-2-phenyl-2-[(3R,4S,5S,6R)-3,4,5-trihydroxy-6-(hydroxymethyl)oxan-2-yl]oxyethanone (peak 7), 1-*O*-methylisocurculigine (peak 9), curculigenin (peak 12), and crassifoside C (peak 17) were all putatively identified based on their mass and mass fragmentations and were compared with available references. Based on the mass fragmentation, the compounds at peaks 4 and 5 were identified as curculigine M and curculigine G; both of which were confirmed by the MN when both the compounds appeared in the same cluster. Lastly, six known norlignans and phenolic glycoside derivatives were identified with all of them visualized in different clusters due to their slightly different structures.

**Table 1:** Annotated compounds based on LC-MS/MS and MN in the rhizome extract of *Curculigo* *latifolia*. The table shows peak number, proposed compound, retention time in minutes (RT), parent ion [M − H]^-^ mass per charge ratio, MS/MS mass per charge ratio of fragment ions, and references.

Peak Number	Retention Time (RT)	[M − H]^−^ (*m*/*z*)	MS/MS (*m*/*z*)	Proposed Compound	Reference
1	5.39	353	353, 191	4-[[3-(3,4- Dihydroxyphenyl)-1-oxo-2-propenyl]oxy]-1,3,5-trihydroxycyclohexanecarboxylic acid.	[27]
2	5.46	495	495, 315, 161	Curculigine	[36]
3	5.61	991	991, 495	Unknown	-
4	5.74	353	353, 155, 135	Curculigine M	[34]
5	5.85	333	333, 315, 109	Curculigine G	[18]
6	6.20	477	477, 109	Curcapicycloside	[25,37]
7	6.46	407	407, 379, 226	1-(2,3-Dihydroxyphenyl)-2-phenyl-2-[(3R,4S,5S,6R)-3,4,5-trihydroxy-6-(hydroxymethyl)oxan-2-yl]oxyethanone	[35]
8	6.63	315	315, 297, 150	Sinensigenin A	[30]
9	6.84	509	509, 311,174	1-*O*-methylisocurculigine	[28,32]
10	6.97	509	509, 371	Breviscaside B	[31]
11	7.04	477	477, 315, 297, 161	Crassifoside D	[29,38]
12	7.50	347	347, 315, 109	Curculigenin	[28]
13	7.65	377	377, 347,174	Unknown	-
14	7.93	347	347, 315	Unknown	-
15	8.11	511	511, 181	Sinenside B	[32]
16	10.58	395	395, 138, 79	Orcinoside J	[26]
17	11.06	493	493, 447, 315, 161	Crassifoside C	[29]

#### 2.2.2. Metabolite Profiling of the Leaf Extract Based on LC-MS/MS and MN

In the leaf extract, ten identified compounds and six unknown compounds were identified and annotated (blue color structure). All ten known metabolites, norlignan metabolites, and phenolic glycosides were successfully identified based on their mass fragmentation pathways, as shown in Table 2 (Figure 2), and all the detected metabolites were compared to published data [27,32,33,34,39,40,41,42,43,44,45]. 

**Figure 8:**
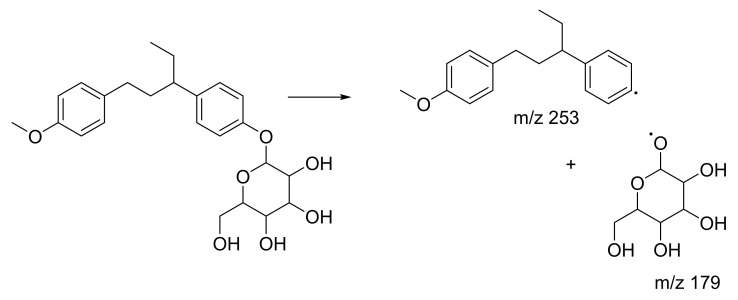
Proposed fragmentation pathway for tetrahydromethylmononyasine A.

Peaks 1, 7, and 12 showed the most abundance in the leaf extract, with all of them identified as phenolic glycoside metabolites. Peak 1 with an RT at 4.35 was identified as tetrahydromethylmononyasine A (Table 2) with the parent ion at *m*/*z* 431 [M − H]^−^ (Figure 8). The parent ion dissociated to yield fragment ions at *m*/*z* 179 because its glucose was eliminated (Supplementary Figure S4).

**Figure 9:**
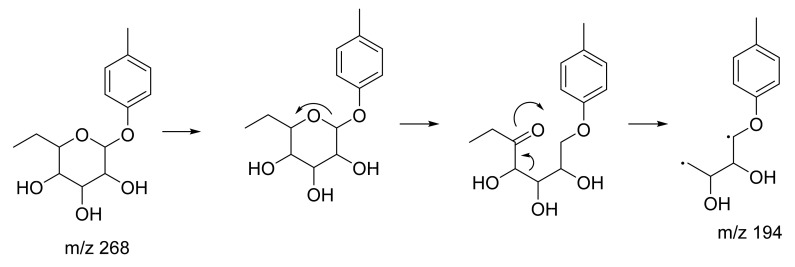
Proposed fragmentation pathway for (2R,4S,5S,6R)-2-ethyl-6-(4-methylphenoxy)oxane-3,4,5-triol.

**Figure 10:**
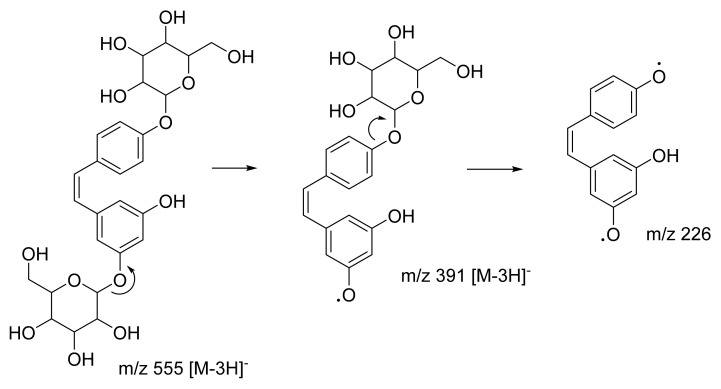
Proposed fragmentation pathway for (Z)-resveratrol 3,4′-diglucoside.

Peak 7 showed characteristics consistent with (*2R,4S,5S,6R*)-2-ethyl-6-(4-methylphenoxy)oxane-3,4,5-triol, with a parent ion at *m*/*z* 267 [M − H]^−^. The peak appeared at RT 12.20, yielding MS/MS fragment ions at *m*/*z* 194. A possible fragmentation pathway for (*2R,4S,5S,6R*)-2-ethyl-6-(4-methylphenoxy)oxane-3,4,5-triol is shown in Figure 9 (Supplementary Figure S5). The third highest abundance is peak 12 with RT 15.79 attributed to (*Z*)-resveratrol 3,4′-diglucoside. In Figure 10, this metabolite exhibited parent ions at *m*/*z* 552 [M − 3H]^−^ and characteristic MS/MS fragment ions at *m*/*z* 389 [M − 3H]^−^ and *m*/*z* 226, indicating a loss of two glucose compounds from the parent metabolite (Supplementary Figure S6). Metabolites at peaks 7 and 12 have never been identified in *C. latifolia* and these fragmentation results agree with that of past studies [40,43]. Even though the compounds at peaks 1, 7, and 12 are phenolic glycosides, they were all visualized in three different clusters in the MN. This is because their structures are all slightly different.

Surprisingly, when annotating the LC-MS/MS analysis data (Figure 2) in MN, at RT 10.84 (*m*/*z* 331) and RT 11.06 (*m*/*z* 293) two high peaks were thought to be attributed to unknown compounds. However, when viewed from the MN, the mass shows an overlap with a blank node (circled in red in Figure 3), therefore the peaks were deduced to be blanks and not compounds from the leaf extract.

In the MN of the leaf extract, two major clusters were observed, one cluster was annotated as phenolic glycosides derivatives and consists of tetrahydromethylmononyasine A (peak 1), 2-(4-ethylbenzyl)-5-hydroxymethylphenyl 4-deoxy-4-C-methyl-beta-D-glucopyranoside (peak 2), orcinosides E (peak 3), and orcinol gentiobioside (peak 4). Another major cluster was unidentified owing to two unknown compounds that have yet to be putatively annotated (peaks 8 and 10). Peak 13, another unknown compound, was located on a single node, thus making identification of the compound difficult. Two unknown compounds (peaks 14 and 16) were suspected to be curculigine M derivatives as they were found in the same cluster as curculigine M (peak 15). Another unknown compound at peak 5 with *m*/*z* 327 [M − H]^−^ could potentially be a crassifogenin D derivative (*m*/*z* 329 [M − H]^−^) due to both the compounds being annotated within the same cluster. Lastly, the MN also showed two identified compounds that were generated from a single node, as shown in Figure 3. These compounds were identified as 3, 4, 5-triacetoxybenzoic acid (peak 9) and sinensigenin B (peak 11), both of which exhibit different skeleton structures.

References from the mass spectroscopic databases (https://gnps.ucsd.edu/ProteoSAFe/libraries.jsp, accessed on 6 June 2021, and https://pubchem.ncbi.nlm.nih.gov/, accessed on 6 June 2021) were used to conduct the putative annotations. The metabolites were identified using a systematic analysis of the fragmentation process and patterns observed in the resulting network. As demonstrated in Figure 3, Table 1 and Table 2, there are 14 clusters in the MN of the rhizome extract and leaf extract, with four major clusters and ten minor clusters. The MN was capable of not only dereplicating the norlignan and phenolic glycoside compounds, but also pointing out related derivatives, of which were described for the first time in *C. latifolia*.

**Table 2:** Annotated compounds based on LC-MS/MS and MN identified in the leaf extract of *Curculigo* *latifolia*. The table shows peak number, proposed compound, retention time in minutes (RT), parent ion [M − H]^−^, mass-per-charge ratio, MS/MS mass per charge ratio of fragment ions, and references.

Peak Number	Retention Time (RT)	[M − H]^−^ (*m*/*z*)	MS/MS (*m*/*z*)	Proposed Compound	Reference
1	4.35	431	431, 385, 289, 179	Tetrahydromethylmononyasine A	[39]
2	5.38	403	403, 223 401, 241, 223	2-(4-Ethylbenzyl)-5-hydroxymethylphenyl 4-deoxy-4-C-methyl-beta-D-glucopyranoside	[44]
3	6.15	415	415, 248, 226, 174	Orcinosides E	[41]
4	7.86	445	445, 384, 174	Orcinol gentiobioside	[27]
5	8.51	327	327, 248, 225, 174	Unknown	-
6	9.07	329	329, 248, 226, 191, 174	(*1R, 2R*)-crassifogenin D or (*1S, 2R*)-crassifogenin D	[33]
7	12.20	267	267, 248, 194,	(*2R,4S,5S,6R*)-2-Ethyl-6-(4-methylphenoxy)oxane-3,4,5-triol	[43]
8	12.90 13.84	723 721	721, 514, 248	Unknown	-
9	13.34 14.99	293	293, 248, 236, 174	3, 4, 5-Triacetoxybenzoic acid	[42]
10	14.11	559	559, 514, 384, 248, 174	Unknown	-
11	14.30 14.87	295 297	295, 190	Sinensigenin B	[32]
12	15.79	555	555, 391, 226	(*Z*)-Resveratrol 3,4′-diglucoside	[40,45]
13	16.99	271	271, 248, 212, 197	Unknown	-
14	17.47, 18.61	377 379	377, 277, 248, 212	Unknown	-
15	19.70	355	355, 299, 226, 194	Curculigine M	[34]
16	19.87	381	381, 349, 281, 226	Unknown	-

### 2.3. The Total Phenolic Content of Methanol Extracts

Total phenolic content (TPC) shows the amount of phenolic content in a sample. The TPC of the *C. latifolia* fruit, leaf, and rhizome extracts were determined using a colorimetric method adapted from Kassim et al. [46] (Folin–Ciocalteu reagent), using gallic acid (GAE) as the standard. In Table 3, of the methanol extracts, the rhizome extract showed the highest TPC, which was 175.75 ± 0.43 GAE (µg/g) of the extract, followed by the fruit extract, at 153.34 ± 0.32 GAE (µg/g). The lowest TPC was detected in the leaf extract, at 130.69 ± 0.48 GAE (µg/g). The TPC data shows the analysis of the free radical-scavenging and reducing properties of the extracts. The higher the TPC value in a sample, the better the redox properties of the sample, indicating good antioxidant properties [47,48]. The higher phenolic content in the rhizome extract is responsible for more bioactivity; therefore, this extract is expected to exhibit good antioxidant and antibacterial activities.

### 2.4. Antioxidant Capacity of Curculigo Latifolia Extract (Fruit, Leaves, and Rhizome)

Knowing that the antioxidant of the plant extract is influenced by pleasure factors, it is important to apply multiple analyses on the antioxidant capacities of the extracts to cover the various mechanisms of antioxidant action [48,49]. Therefore, in this study, the extracts of *C. latifolia* were examined using two antioxidant assays: a DPPH^•^ radical scavenging assay and a beta-carotene assay. DPPH^•^ is a stable free radical and is commonly employed to assess the radical scavenging activity of plant extracts. Antioxidant molecules can quench the DPPH^•^ radical (by providing a hydrogen atom or electron donation) and can convert the radical to a colorless product [50]. Meanwhile, in the beta-carotene assay, the antioxidant activity coefficient (AAC) was measured in terms of the successful bleaching of β-carotene. β-Carotene undergoes a rapid discoloration in the absence of an antioxidant. Antioxidant activity was determined by measuring the inhibition of organic compounds and the conjugated diene hydroperoxides arising from linoleic acid oxidation [51,52].

#### 2.4.1. DPPH^•^ Radical Scavenging Activity Assay

Table 3 shows that the rhizome extract of *C. latifolia* has considerably higher DPPH^•^ radical scavenging activities compared to the ascorbic acid (vitamin C) standard. In contrast, the fruit and leaf extracts exhibit low DPPH^•^ radical scavenging activities. As for the IC_50_ (µg/mL) (Table 3), the rhizome extract exhibited the lowest IC_50_ value at 18.10 ± 0.91 µg/mL, followed by the fruit extract at 26.99 ± 1.58 µg/mL. Meanwhile, the highest IC_50_ was observed in the leaf extract at 547.29 ± 5.09 µg/mL. As lower IC_50_ values indicate higher antioxidant activity, the rhizome extract had the highest ability to scavenge free radicals compared to the fruit and leaf extracts. The high antioxidant activity as demonstrated by the DPPH^•^ radical assay of the rhizome extract mirrors the results of the TPC analysis.

#### 2.4.2. Determination of Antioxidant Activity via the β-Carotene Bleaching Method

In the β-carotene bleaching assay, linoleic acid produces hydroperoxides as free radicals during incubation at 50 °C and attacks the β-carotene molecules; in turn, causing a reduction in the absorbance at 470 nm. Butylated hydroxyanisole (BHA), butylated hydroxytoluene (BHT), vitamin C, and α-tocopherol (vitamin E) were used as the standard. In the system, β-carotene will undergo rapid discoloration in the absence of antioxidant content in the extract and vice versa in the presence of antioxidant content [2,52]. The rapid discoloration of β-carotene–linoleate depends on the antioxidant content of the extracts. Hence, the presence of different antioxidants can delay the extent of the β-carotene bleaching by neutralizing the linoleate free radicals and other free radicals formed in the system. In Table 3, of the *C. latifolia* extracts, the rhizome extract showed the highest inhibition among the extracts of antioxidant activity against β-carotene-linoleic acid, and by the positive control (BHT, BHA, vitamin E, and vitamin C). The result showed a considerable variation in the antioxidant activities ranging from 28.55% to 35.2%, where the orders of the antioxidant activity are as follows: rhizomes > leaves > fruit. Unfortunately, the order of antioxidant activity of *C. latifolia* extracts in β-carotene (rhizome > leaves > fruit) was not the same as that of the DPPH^•^ and TPC (rhizome > fruit > leaves). Therefore, it is obvious that the TPC of the fruit (Table 3) has a stronger effect against scavenging activity compared to the discoloration of β-carotene [53].

Table 3 highlights a positive correlation between TPC and antioxidant activity based on the DPPH^•^ assay, where the order of the antioxidant activity is as follows: rhizomes > fruit > leaves. Conversely, no correlation was found for the antioxidant activity based on the β-carotene bleaching assay with TPC. This result is in agreement with Maisarah et al. [53], who also found no relationship between the β-carotene bleaching activity and the total phenolic content in *Carica papaya* extracts (ripe and unripe fruit, seeds, and young leaves). Lastly, this result provides a basis for the further exploration of the antioxidant components of the *C. latifolia* plant via LC-MS/MS analysis and MN visualization.

### 2.5. Antimicrobial Assay

Table 4 summarizes the antibacterial activity exhibited by the rhizome, leaf, and fruit extracts against *S. choleraesuis* (Gram-negative bacteria) and *S. aureus* (Gram-positive bacteria). The leaf extract showed the highest antibacterial activity towards *S. aureus ATCC 43300 and S. choleraesuis ATCC 10708*, producing inhibition zones of ±15.33 mm and ±8 mm respectively. Meanwhile, the fruit extract showed smaller inhibition zones of ±8.00 mm against *S. aureus ATCC 43300*, suggesting that the fruit extract either contains different bioactive constituents or lower amounts of the same bioactive constituents [54]. The rhizome extract showed no to weak activity against the indicator microorganisms. Studies have been conducted to investigate the efficacy of plant extracts and their active compounds as antibacterial agents, suggesting that the bioactive component(s) of plant extracts interact with enzymes and proteins of the bacterial cell membrane, which may cause (i) disruption of the cell membrane, resulting in a flux of protons towards the cell exterior and ultimately causing cell death, or (ii) inhibition of enzymes that are required for amino acid biosynthesis [55].

Venkatachalam et al. [9] investigated the antibacterial properties of the *C. orchioides* leaf extracts. They reported that the leaf extract exhibited the highest activity against *P. aeruginosa* (18 mm) and *S. aureus* (14 mm). Yadav and Shukla [54] reported that *C. orchioides* exhibited antibacterial activity against the bacterial strains *Escherichia* *coli, P. aeruginosa, Streptococcus pyogenes,* and *S. aureus*. The presence of pharmaceutically active compounds like 3′,8,8′-Trimethoxy-3-piperidyl-2,2′-binaphthalene-1,1′,4,4′-tetrone; paromomycin; geranyl isovalerate; tert-hexadecanethiol; 1,2-Propanediol; 3-(tetradecyloxy); n-hexadecanoic acid; (2-phenyl-1,3-dioxolan-4-yl)methyl ester;6-[1-(acetyloxy)-3-oxobutyl] -3,3a,4,7,8,8a-hexahydro-7-methyl-3-methylene; d-Lyxo-d-manno-nononic-1,4-lactone; 7-methyl-ztetradecen-1-ol acetate; 9-octadecenoic acid and trans and 2H-cyclohepta [b] furan-2-one have been shown to affect significant activity against bacterial pathogens. Overall, these results suggests that the *Curculigo* plant could be utilized as active ingredients in antibacterial products due to their potential antimicrobial activity.

Because the leaf extract has better antimicrobial effects on the two tested strains, the leaf extract was subjected to MIC and MBC against *S. aureus* and *S. choleraesuis*. The comparative data are listed in Table 4 and Table 5. The leaf extract was found to be more potent against the two standard strains, i.e., *S. aureus* (MIC ± 0.25 M; MBC ± 0.25 M) and *S. choleraesuis* (MIC ± 0.25 M; MBC ± 0.25 M).

The leaf extract antibacterial activity may be due to the presence of active phenolic compounds such as phenols and phenolic glycosides [5]. Hence, the leaf extract was analyzed via LC-MS/MS to profile the metabolite. Among the extracts, the leaf extract exhibited three high abundances which were identified as tetrahydromethylmononyasine, (*2R,4S,5S,6R*)-2-ethyl-6-(4-methylphenoxy)oxane-3,4,5-triol, and (*Z*)-resveratrol 3,4′-diglucoside, respectively. Presently, no literature exists that shows that these compounds are responsible for any antibacterial activity, therefore an isolation of these three compounds should be performed in order to confirm any form of antibacterial activity. The findings of the current study endorse the traditional usage of medicinal plants in medical treatment and recommends the usage of plant extracts with antibacterial properties as antibacterial agents.

## 3. Materials and Methods

### 3.1. Sample Collection

The leaf, fruit, and rhizomes of *C. latifolia* were collected locally from Kuala Pilah, Negeri Sembilan, Malaysia. The fruits (15.17 g) were collected in polyethylene bags and stored at 4 °C. The fruit samples were immediately ground into a fine powder using mortar and pestle in liquid nitrogen and then freeze-dried and stored at −80 °C until further use. The leaf (75.36 g) and rhizome (42.09 g) samples were washed, cut, air-dried, ground to fine powder, and stored at room temperature before extraction (Supplementary Figure S7). Voucher specimens of the samples were deposited at the Institute of Bioscience, Universiti Putra Malaysia, for future reference (SK 3370/18).

### 3.2. Extraction

#### 3.2.1. Fruits

The freeze-dried fruit sample was ground into a fine powder and immersed in 100 mL of 70% methanol in an amber bottle. The amber bottle was shaken to mix the powder with the solvent, and the mixtures were ultrasonicated for one hour at room temperature. The extraction procedure was performed twice on the samples, and the extract was concentrated under reduced pressure using a rotary evaporator. The concentrated extract was stored in amber bottles, which were placed in a chiller for future use in biological assays.

#### 3.2.2. Leaf and Rhizomes

The powder of the leaf and rhizomes were soaked in 100% methanol (750 mL) for three days at room temperature. The supernatant was then collected, filtered, and the solvent evaporated using a vacuum rotary evaporator. This step was done twice. The crude extracts were stored at room temperature to determine their antioxidant and antimicrobial activities.

### 3.3. Ultra-High Performance Liquid Chromatography–MS/MS (UHPLC-MS/MS) Analysis

The samples were prepared using a method described by Oliveira et al. [56] The crude methanol extract was separated using a C18 Reversed-phase Hypersil GOLD aQ column (100 × 2.1 mm ~1.9 µm) (Thermo, Waltham, MA, USA) at 30 °C on a Dionex Ultimate 3000 UHPLC with a diode-array DAD-3000 detector (Thermo Fisher Scientific, Waltham, MA, USA). The UHPLC-MS/MS analyses were conducted based on a method described by Buzgaia et al. [4]. The MS data analyses were conducted using the ThermoXcalibur 2.2 SP1.48 software (Thermo Fisher Inc. Waltham, MA, USA) and based on readily available literature data. The MS data were firstly converted into the mzXML format using the MSConvert software. The generated spectral information was then uploaded to MZmine-2.5.3-Windows to generate the MS/MS data [57,58].

### 3.4. Molecular Networking (MN)

The MS and MS/MS data of *C. latifolia* was subjected to MN analysis. The mass spectroscopic data were transformed into the mzXML format [59], and then processed on an online platform (http://gnps.ucsd.edu, accessed on 21 August 2019) according to a MN method [60]. The detailed metabolite information of the crude extract can be accessed via https://gnps.ucsd.edu/ProteoSAFe/libraries.jsp, accessed on 6 June 2021 and https://pubchem.ncbi.nlm.nih.gov/, accessed on 6 June 2021 (crude extract + references). The spectral networks were imported using the Cytoscape 3.7.1 software and visualized using a force-directed layout.

### 3.5. Antioxidant Assay

The antioxidant activities of the *C. Latifolia* extracts and fractions were estimated using DPPH assays, TPC assays, and *β*-carotene bleaching assays.

#### 3.5.1. 2,2-Diphenyl-1-picryl-hydrazyl-hydrate (DPPH^•^) Assay

The DPPH^•^ scavenging activity was determined following a method described by Kassim et al. [46] with slight modifications. Vitamin C was used as the standard. The scavenging percentages of the samples were obtained using the formula:

% Inhibition = 1 − (OD_sample_/OD_blank_) × 100

where OD_blank_ is the blank absorbance and OD_sample_ is the reaction mixture absorbance. By plotting the DPPH scavenging percentage against the sample concentration, the IC_50_ value was subsequently determined.

#### 3.5.2. Total Phenolic Content (TPC)

The phenolic content of the samples was determined using Folin–Ciocalteu’s reagent adapted from a method described by Kassim et al. [46]. A calibration graph of various concentrations of gallic acid was constructed as the standard. The TPC was represented by the mg GAE of dry weight.

#### 3.5.3. β-Carotene Bleaching Assays

The β-carotene bleaching assay [46] was used to measure the antioxidant properties of the crude extracts and fractions. BHA, BHT, vitamin C, and vitamin E were used as the standard. The absorbance reading of the samples was taken at the start (0 min) and also at the end after 120 min of incubation. The antioxidant activity (AA) was determined according to the formula:

AA% = 1 − [(A_t=0_ − A_t=120_)/(A_C=0_ − A_C=120_)] × 100

where A_t=0_ and A_t=120_ are the absorbance of the test samples measured at 0 min and 120 min, respectively; and A_C=0_ and A_C=120_ are the absorbances of the control measured at 0 min and 120 min, respectively.

### 3.6. Antimicrobial Screening of Extracts

#### 3.6.1. Microorganisms

Two types of Gram-negative bacteria (*S. choleraesuis* ATCC 10708 and *E.* *coli* ATCC 25922) and two types of Gram-positive bacteria (*Bacillus subtilis* B29 and *S. aureus* ATCC 43300) were used for the spectrum antimicrobial study. The cultures of the microorganisms were obtained from Institute of Bioscience, Universiti Putra Malaysia.

#### 3.6.2. Antibacterial Assay Methodology (Disk Diffusion Assay)

The antibacterial activity was evaluated using an agar diffusion assay, as described by Buzgaia et al. [61]. Positive (Streptomycin (100 mg/mL), Streptomycin sulfate–BIO BASIC INC, Canada) and negative (dimethyl sulfoxide, 10% DMSO, Merck, Darmstadt, Germany) control discs were similarly prepared and placed on each test plate. All experiments were conducted in triplicates, and the inhibition zone diameter (IZD) was measured in mm.

#### 3.6.3. Determination of Minimum Inhibitory Concentration (MIC) and Minimum Bactericidal Concentration (MBC) Values

The MIC and MBC values of the test samples against bacteria were determined as described by the Institute of Bioscience, Universiti Putra Malaysia. The analysis was performed in a 96-well round-bottom microtiter plate (Greiner, Germany) using a 2-fold standard broth microdilution method with an inoculum of about 106 CFU/mL. The MIC value is defined as the lowest concentration of the test sample that completely inhibits bacterial growth. To determine the MBC value, a 10 µL aliquot of the suspension in each of the 12 wells of the MIC determination was subcultured on a Mueller Hinton Agar (MHA) plate. The MBC is defined as the lowest concentration of the test sample that completely kills the bacterial strain. The MIC and MBC values were determined in duplicates. Streptomycin (Streptomycin sulfate—BIO BASIC INC, Canada) was used as the positive control.

## 4. Conclusions

In this study, the LC-MS/MS and MN approaches were combined and developed to target and identify active compounds in a crude extract. This method was applied on the *C. latifolia* rhizome and leaf extracts to highlight and identify the metabolites responsible for the plant’s antioxidant and antimicrobial capabilities. This is the first known report for *C. latifolia* that utilizes an MN visualization based on a LC-MS/MS spectra that shows norlignans and phenolic glycosides as major families in the crude extract. The crude extracts were then analyzed for their antioxidant and antimicrobial activities. From the TPC, DPPH, and beta-carotene antioxidant analyses, it was shown that the rhizome extract is rich in TPC and has the best antioxidant activity among the extracts performed in this study. As such, the rhizome of *C. latifolia* could be considered a bioactive functional plant with high phenolic content and remarkable antioxidant activity. Meanwhile, the leaf extract demonstrated significant antimicrobial activity against the tested microorganisms, including *S. choleraesuis* (Gram-negative bacteria) and *S. aureus* (Gram-positive bacteria), exhibiting the widest zone of inhibition. The results of this study confirm the therapeutic potency of the *Curculigo* plant, and scientifically validates the effectiveness of the medicinal plant to treat infectious diseases in a local and traditional manner. However, the mechanism of their actions should be further elucidated; in particular, the actual constituents responsible for bioactivity should be isolated, identified, purified, and structurally clarified.

## Figures and Tables

**Table 3:** Antioxidant activity of the *Curculigo latifolia* crude extract.

	DPPH^•^ IC_50_ (µg/mL)	TPC GAE (µg/g)	β-Carotene (%)
**Fruit**	26.99 ± 1.58	153.34 ± 0.32	28.55 ± 4.17
**Rhizome**	18.10 ± 0.91	175.75 ± 0.43	35.20 ± 4.74
**Leaf**	547.29 ± 5.09	130.69 ± 0.48	31.38 ± 0.60
**Vitamin C**	11.49 ± 0.071	-	52.71 ± 5.25
**Vitamin E**		-	93.35 ± 0.30
**BHA**		-	80.56 ± 2.78
**BHT**		-	77.76 ± 0.80

All values are expressed as mean ± standard deviation of triplicates.

**Table 4:** Inhibition zones of *Curculigo latifolia* methanolic extracts against *Bacillus subtilis* B29, *Staphylococcus aureus* ATCC 43300, *Escherichia coli* UPMC 25922, and *Salmonella choleraesuis* ATCC 10708.

Sample	Target Microbe											
	*Bacillus subtilis B29*			*Staphylococcus aureus ATCC 43300*			*Escherichia coli UPMC 25922*			*Salmonella choleraesuis ATCC 10708*		
	Inhibition Zone (mm)			Inhibition Zone (mm)			Inhibition Zone (mm)			Inhibition Zone (mm)		
	i	ii	iii	i	ii	iii	i	ii	iii	i	ii	iii
Fruit	-	-	-	7	8	7	-	-	-	-	-	-
Leaf	-	-	-	15	16	15	-	-	-	8	8	8
Rhizome	-	-	-	-	-	-	-	-	-	-	-	-
Streptomycin (+ve)	29			30			25			32		

**Table 5:** Minimum inhibitory concentration (MIC) and minimum bactericidal concentration (MBC) values (mg/mL) of the leaf methanolic extract of *Curculigo* *latifolia* against *Staphylococcus aureus* ATCC 43300 and *Salmonella choleraesuis* ATCC 10708.

Experimental Strain	Leaf (1.0 mg/mL)	
	Minimum Inhibitory Concentration, MIC (mg/mL)	Minimum Bactericidal Concentration, MBC (mg/mL)
*Staphylococcus aureus*	±0.25	±0.25
*Salmonella choleraesuis*	±0.25	±0.25

## Data Availability

Data is contained within the article.

## Supplementary Materials

The following are available online at https://www.mdpi.com/article/10.3390/plants10081488/s1, Figure S1. Proposed fragmentation pathway for 4-[[3-(3,4-Dihydroxyphenyl)-1-oxo-2-propenyl]oxy]-1,3,5-trihydroxycyclohexanecarboxylic acid; Figure S2. Proposed fragmentation pathway for Sinensigenin A; Figure S3. Proposed fragmentation pathway for Curculigine; Figure S4. Proposed fragmentation pathway for Dimer of Curculigine; Figure S5. Proposed fragmentation pathway for Tetrahydromethylmononyasine A; Figure S6. Proposed fragmentation pathway for (2R,4S,5S,6R)-2-Ethyl-6-(4-methylphenoxy)oxane-3,4,5-triol; Figure S7. Proposed fragmentation pathway for (Z)-Resveratrol 3,4′-diglucoside.
