# Supplementary material for: Combination of Molecular Networking and LC-MS/MS Profiling in Investigating the Interrelationships between the Antioxidant and Antimicrobial Properties of Curculigo latifolia
Source: Plants (Basel). 2021 Jul 21;10(8):1488. doi: 10.3390/plants10081488 (PMC8401502; doi:10.3390/plants10081488)
Supplement: Supplementary file 1 [file plants-10-01488-s001.zip › plants-1256522-supplementary.pdf]

Supplementary Figures S1

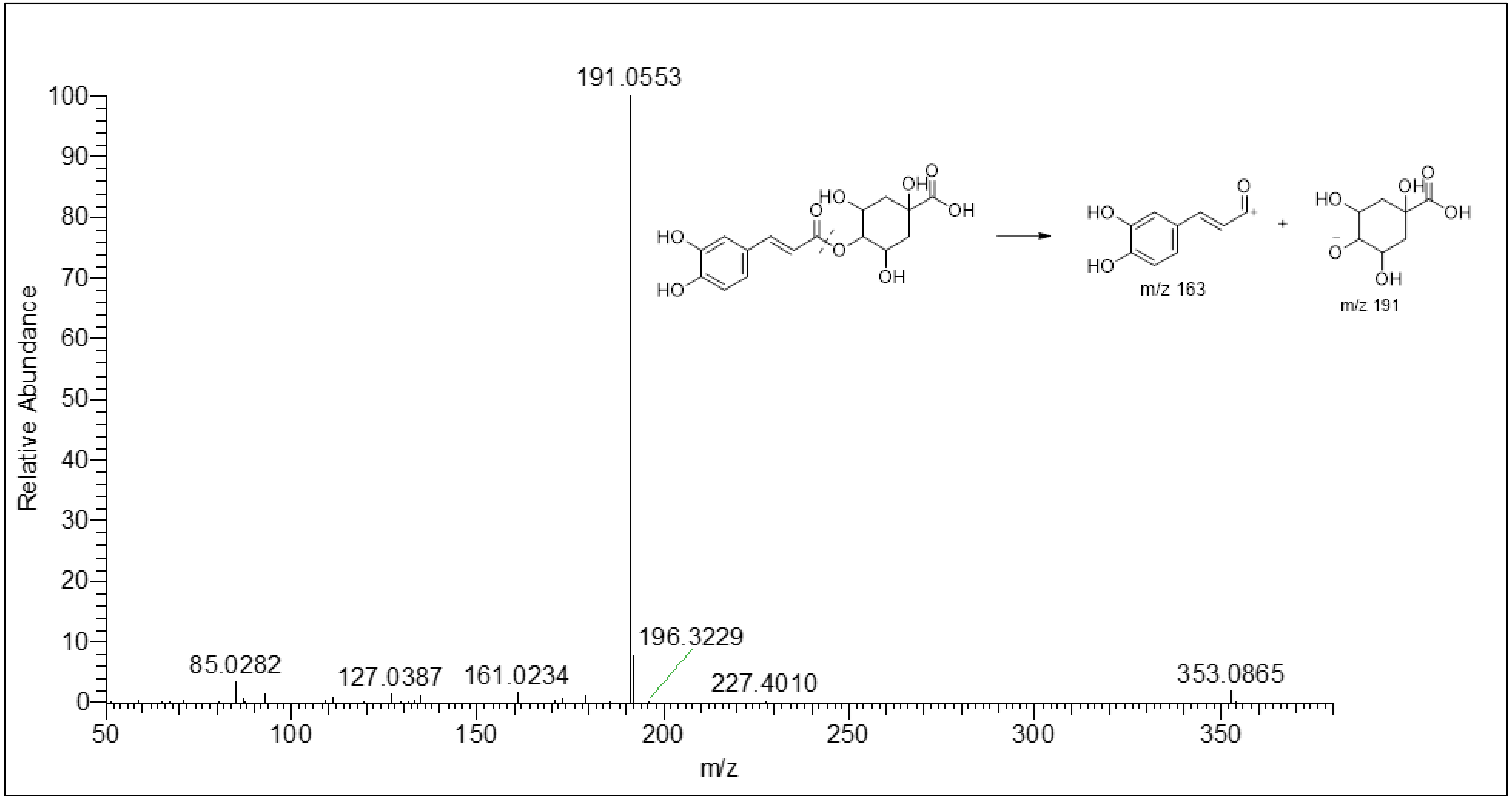

Figure S1. Proposed fragmentation pathway for 4-[[3-(3,4- Dihydroxyphenyl)- 1-oxo-2- propenyl]oxy]- 1,3,5-trihydroxycyclohexanecarboxylic acid.

Supplementary Figures S2

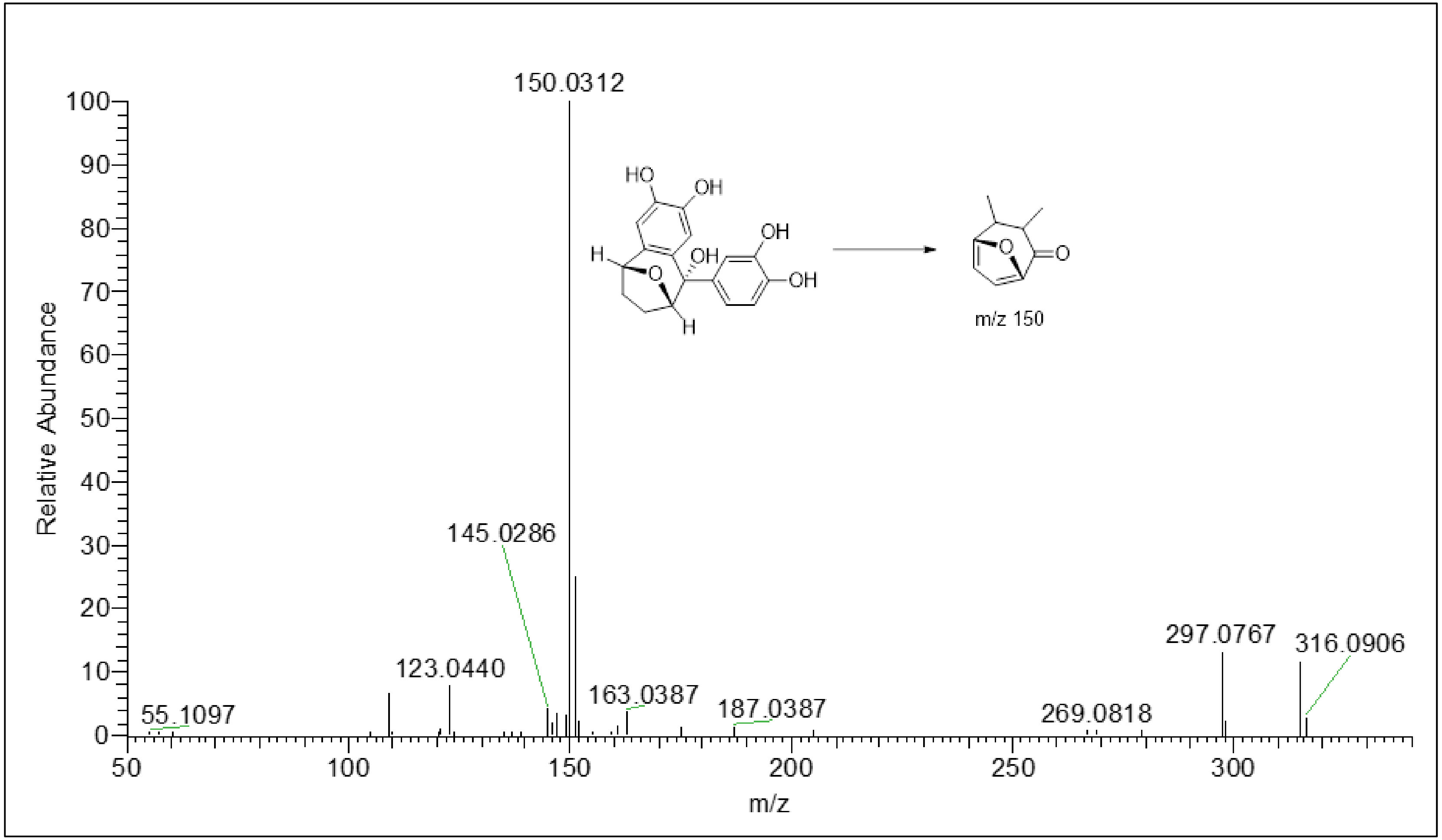

Figure S2. Proposed fragmentation pathway for Sinensigenin A.

Supplementary Figures S3

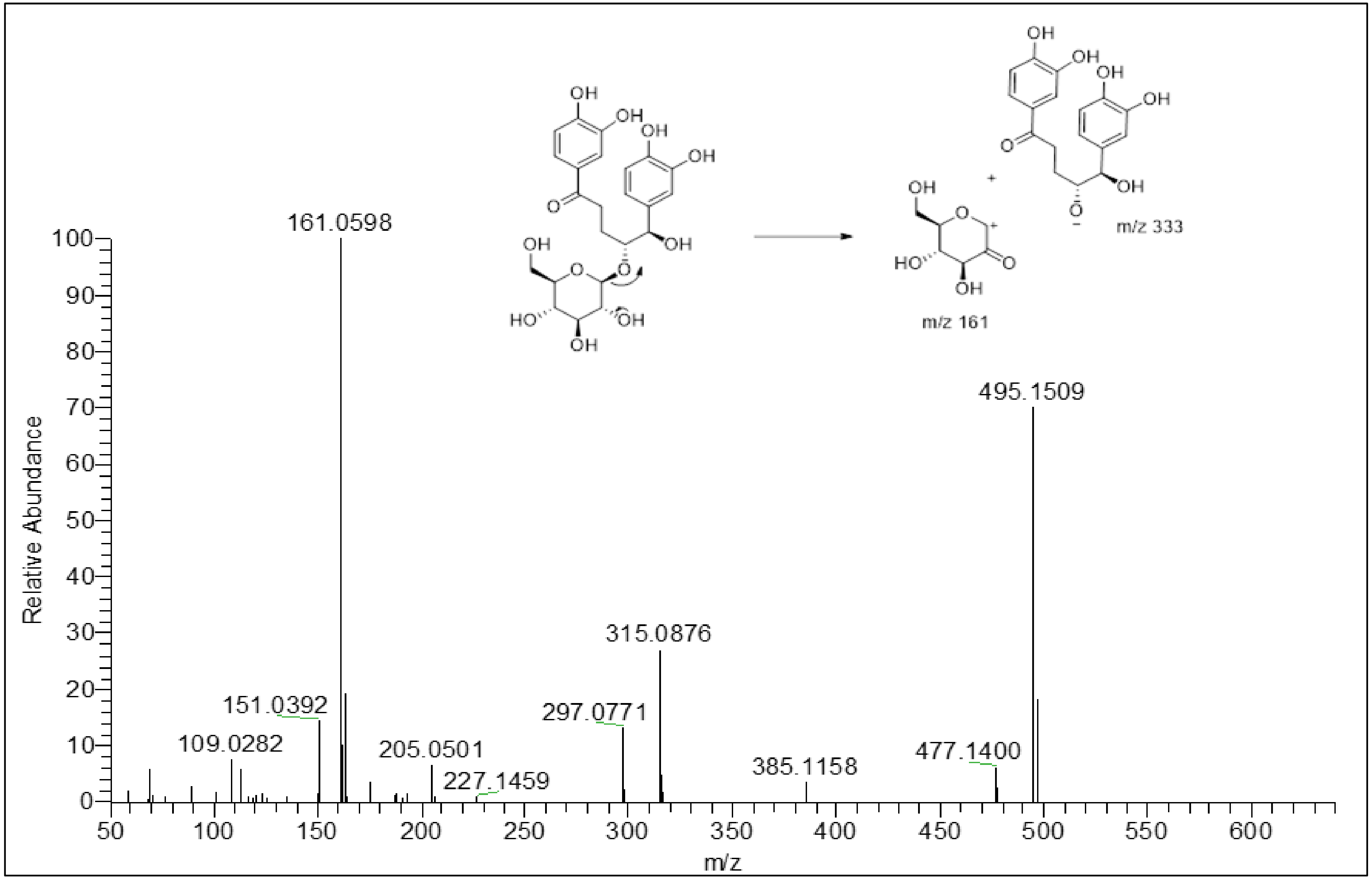

Figure S3. Proposed fragmentation pathway for Curculigine.

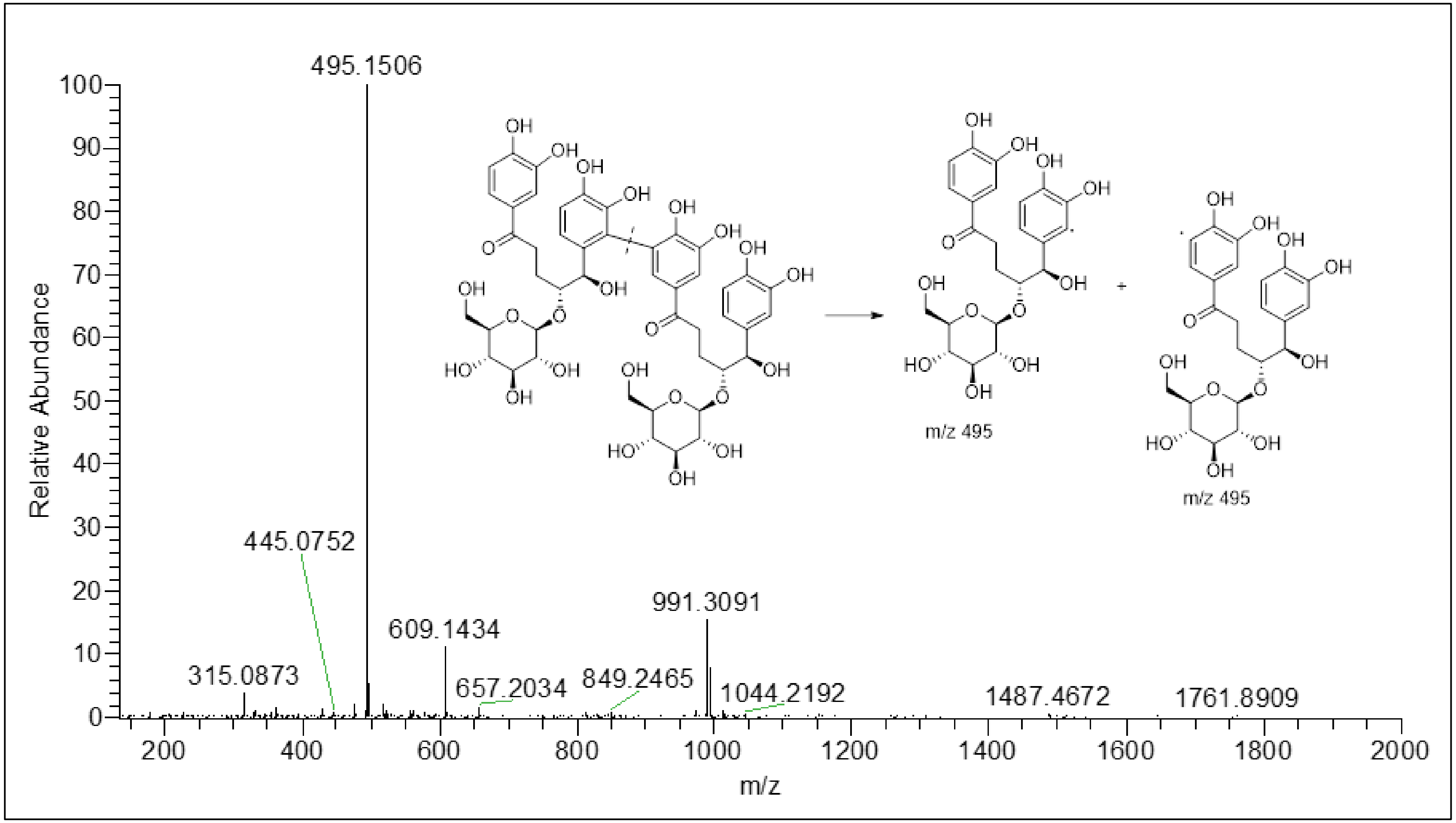

Figure S4. Proposed fragmentation pathway for Dimer of Curculigine.

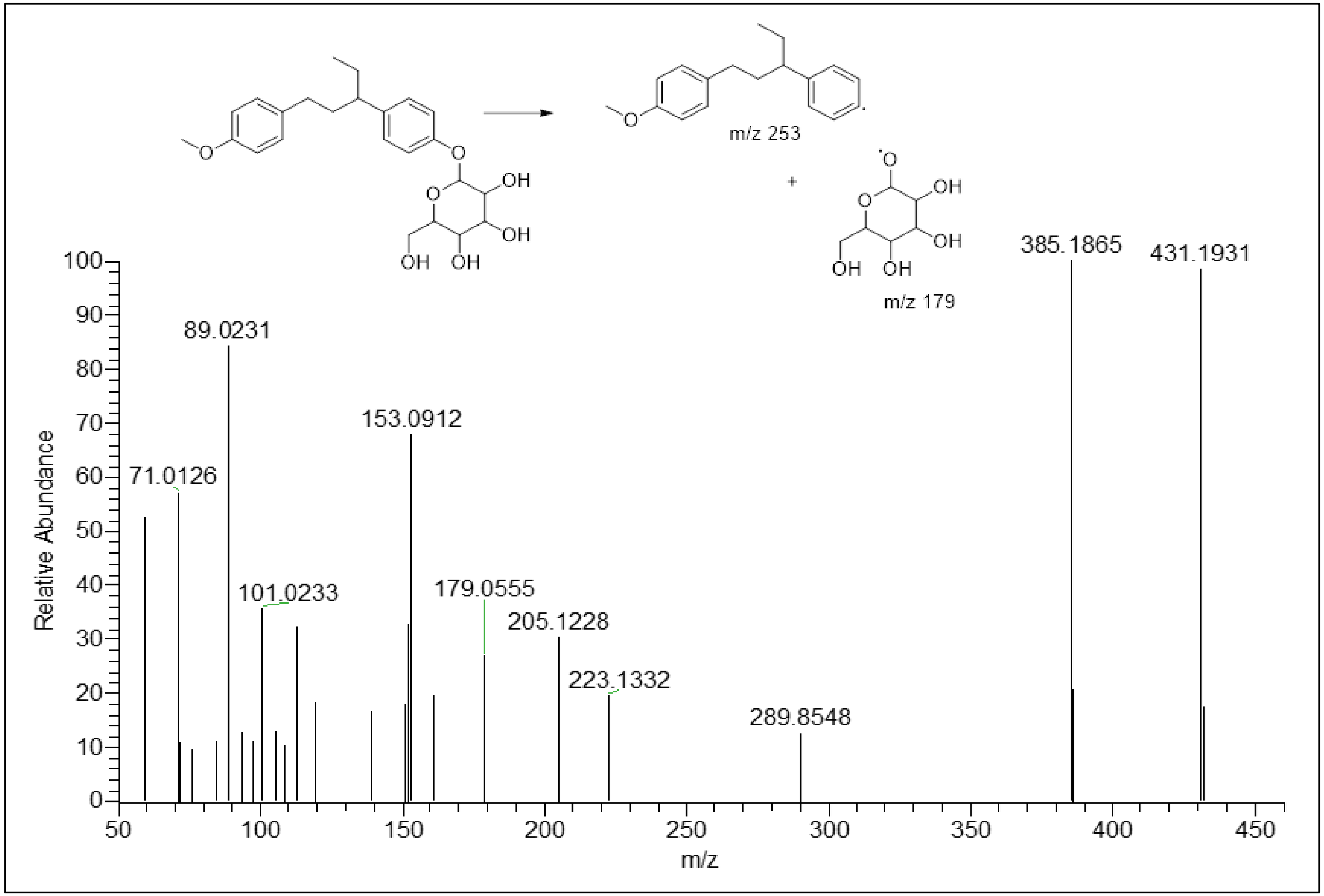

Figure S5. Proposed fragmentation pathway for Tetrahydromethylmononyasine A.

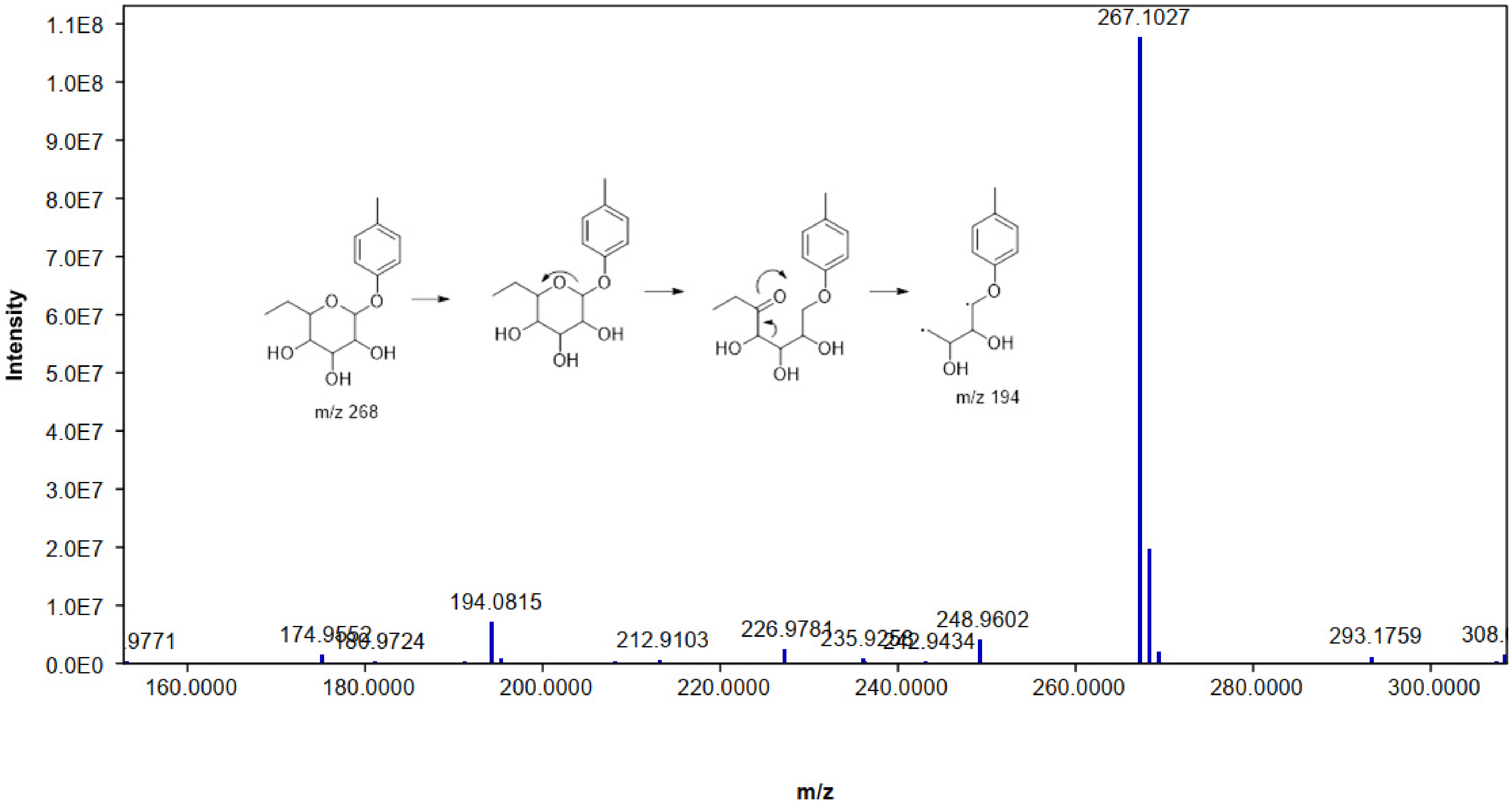

Figure S6. Proposed fragmentation pathway for (2R,4S,5S,6R)-2-Ethyl-6-(4-methylphenoxy)oxane-3,4,5-triol.

Supplementary Figures S6

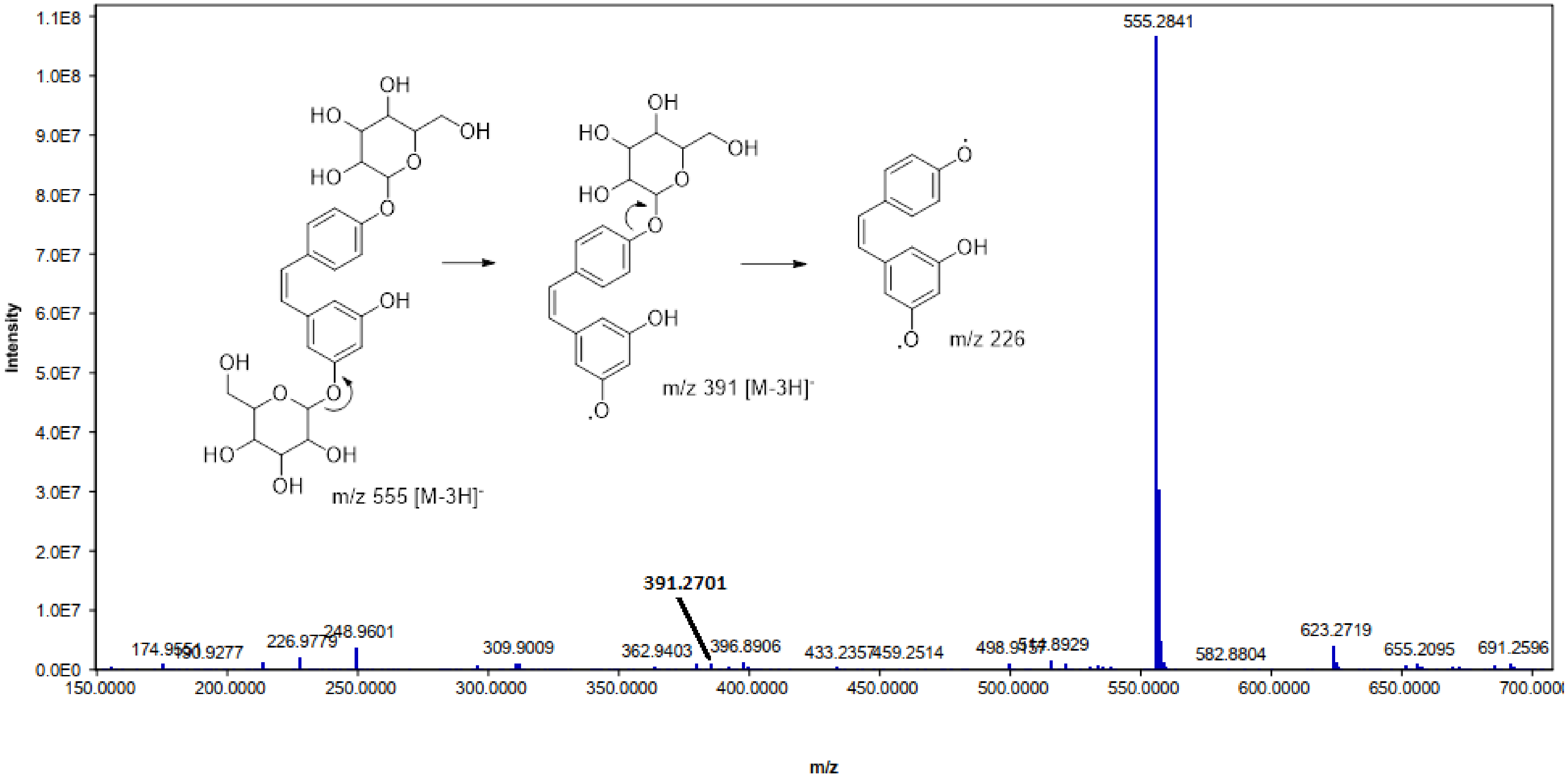

Figure S7. Proposed fragmentation pathway for (Z)-Resveratrol 3,4′-diglucoside.

Figure S8

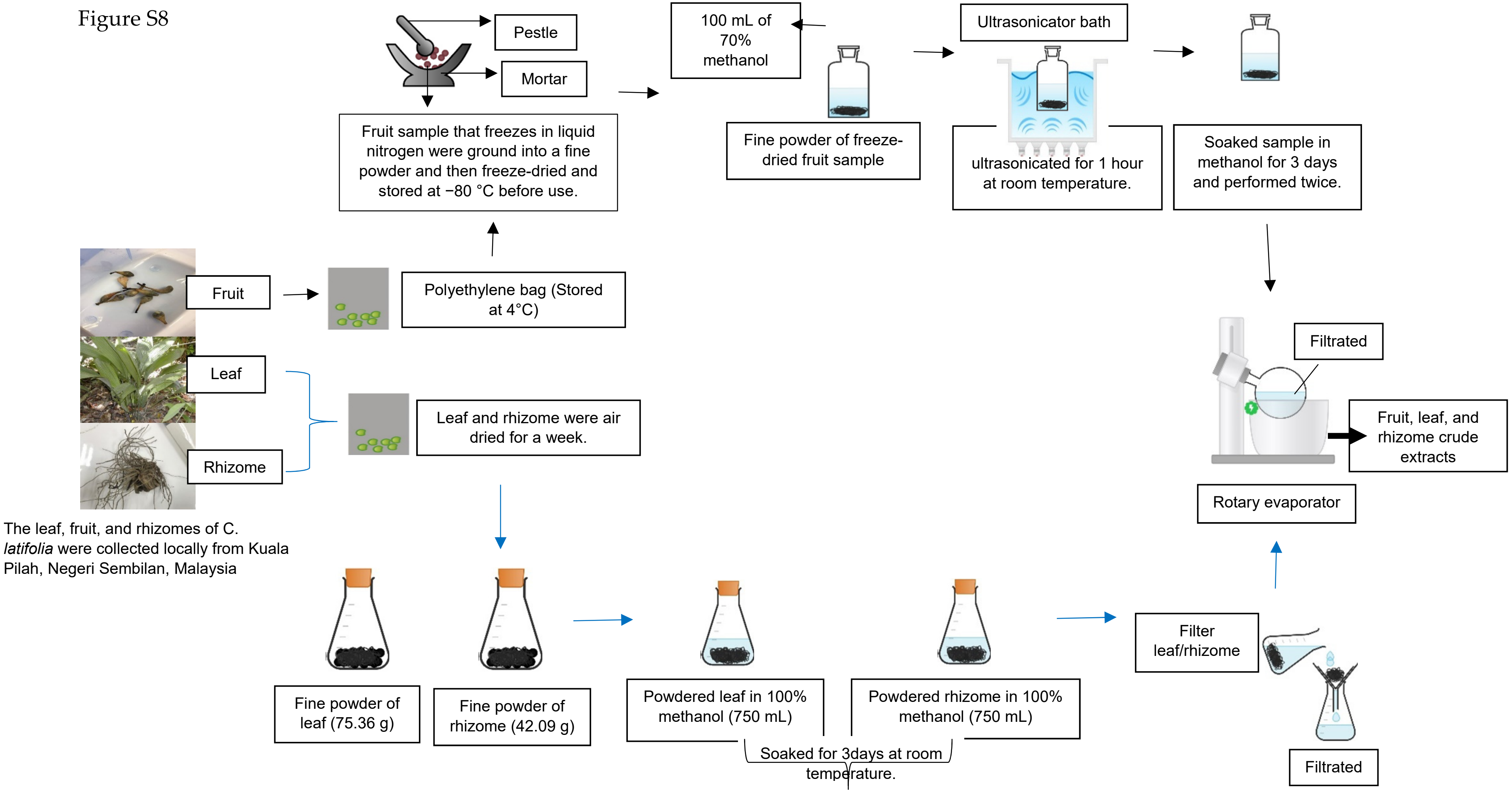

Pestle
Mortar
Fruit sample that freezes in liquid nitrogen were ground into a fine powder and then freeze-dried and stored at −80 °C before use.
100 mL of 70% methanol
Fine powder of freeze-dried fruit sample
Ultrasonicator bath
ultrasonicated for 1 hour at room temperature.
Soaked sample in methanol for 3 days and performed twice.
Fruit
Leaf
Rhizome
Polyethylene bag (Stored at 4°C)
Leaf and rhizome were air dried for a week.
The leaf, fruit, and rhizomes of C. *latifolia* were collected locally from Kuala Pilah, Negeri Sembilan, Malaysia
Filtrated
Fruit, leaf, and rhizome crude extracts
Rotary evaporator
Fine powder of leaf (75.36 g)
Fine powder of rhizome (42.09 g)
Powdered leaf in 100% methanol (750 mL)
Powdered rhizome in 100% methanol (750 mL)
Soaked for 3days at room temperature.
Filter leaf/rhizome
Filtrated
